# 1,3-Cyclohexadien-1-Als: Synthesis, Reactivity and Bioactivities

**DOI:** 10.3390/molecules26061772

**Published:** 2021-03-22

**Authors:** Ignacio E. Tobal, Rocío Bautista, David Diez, Narciso M. Garrido, Pilar García-García

**Affiliations:** 1 Departamento de Química Orgánica, Facultad de Ciencias Químicas, University of Salamanca, Plaza de los Caídos 1–5, 37008 Salamanca, Spain; ignaciotobal@usal.es (I.E.T.); rociobh@usal.es (R.B.); ddm@usal.es (D.D.); nmg@usal.es (N.M.G.); 2 Departamento de Ciencias Farmacéuticas, Facultad de Farmacia, CIETUS, IBSAL, University of Salamanca, Campus Miguel de Unamuno, 37007 Salamanca, Spain

**Keywords:** Diels–Alder, dienes, aldehydes, chiral compounds, synthesis

## Abstract

In synthetic organic chemistry, there are very useful basic compounds known as building blocks. One of the main reactions wherein they are applied for the synthesis of complex molecules is the Diels–Alder cycloaddition. This reaction is between a diene and a dienophile. Among the most important dienes are the cyclic dienes, as they facilitate the reaction. This review considers the synthesis and reactivity of one of these dienes with special characteristics—it is cyclic and has an electron withdrawing group. This building block has been used for the synthesis of biologically active compounds and is present in natural compounds with interesting properties.

## 1. Introduction

One of the reactions that is in the heart of every organic chemist is the Diels–Alder reaction [1]. Dienes are one of the essential components to form the adduct. The reactivity of these compounds in the Diels–Alder reaction varies with electron-withdrawing or donating groups and with conformation restriction as they are part of a cyclic system.

**Figure 1:**
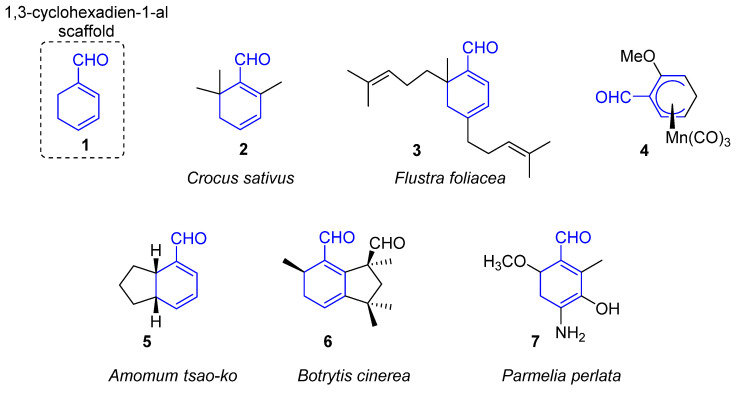
Natural and synthetic compounds with 1,3-cyclohexadien-1-al scaffold.

A very special building block is the 1,3-cyclohexadien-1-al. This scaffold is present in natural products isolated from several plants [2,3], such as 2,6,6-trimethyl-1,3-cyclohexadien-1-carboxaldehyde known as safranal **2** that is the main constituent of the essential volatile oil of *Crocus sativus* L. (saffron) [4], or citral dimer **3**, which has been isolated from marine bryozoan *Flustra foliacea* [5]. Some other examples are compound **5**, extracted from black cardamom (*Amomum tsao-ko*) [6]; compound **6**, which is obtained from the fungus *Botrytis cinerea* [7]; and compound **7** from an Indian fungi *Parmelia perlata* of Parmelia genus [8], which is used as a food supplement. Moreover several synthetic chiral structures such as compound **4** have been obtained [9] (Figure 1). In particular, safranal **2** can be considered a reference compound containing the 1,3-cyclohexadiene-1-al scaffold showing interesting bioactivities, from neuroprotection [10] to anticancer effects [11]. In this review, aromatic aldehydes were not considered due to the different nature of benzaldehydes and related aromatic derivatives in terms of obtention and reactivity.

## 2. Synthesis of 1,3-Cyclohexadien-1-Al Scaffold

There are limited methodologies applied to afford 1,3-cyclohexadien-1-al scaffold. Among them, organocatalytic methods are the more versatile ones, creating the 1,3-cyclohexadienal scaffold through one-pot reactions compared with other methodologies using Lewis acids or formylating agents where the reactant already shows the cyclic 1,3-diene or at least the cyclic system.

**Figure 2:**
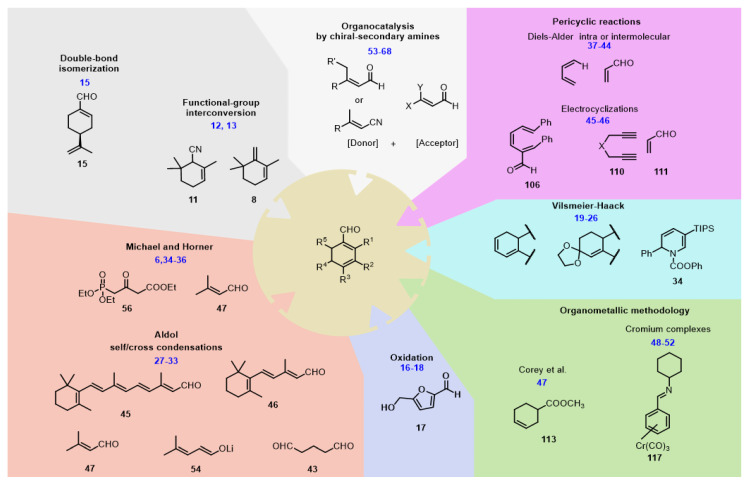
Summary of methodologies for the obtention of 1,3-cyclohexadien-1-als. The corresponding bibliography is shown in blue numbers.

Herein, we review the reported methodologies applied to the synthesis of 1,3-cyclohexadien-1-als. The described methods can be classified as aldol-type condensations, pericyclic cyclizations, functional group interconversions and transformations, and organometallic and organocatalyzed reactions. The general accessibility to this scaffold by the different methodologies is shown in Figure 2.

### 2.1. Functional Group Interconversions and Isomerizations

**Scheme 1:**
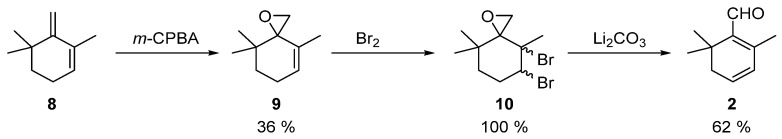
Semisynthesis of safranal **2**.

The simple functional group interconversions can be a pathway for the obtention of 1,3-cyclohexadienals, as in the case of the semisynthesis of safranal **2** starting from γ-pyronene **8** [12] (Scheme 1). The epoxidation of **8** results in a mixture of mono and diepoxide derivatives. The isolated exocyclic epoxide **9** (36%) is transformed in two steps into safranal **2**. Firstly, halogenation of double bound conducts to **10** quantitatively and then a one-pot reaction consisting in the dibromo elimination and epoxide-opening, allowing the obtention of the 1,3-diene system, wherein the hydroxyl group of the intermediate is oxidized, resulting in safranal **2** in good yield.

**Scheme 2:**
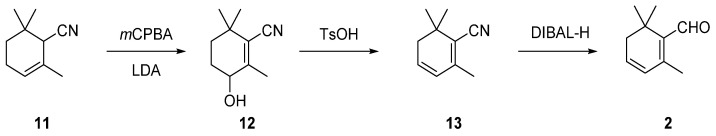
Synthesis of safranal **2** from α-cyclocitronitrile.

Other semisynthesis of safranal **2** starts from α-cyclocitronitrile and takes place in three steps: oxidation, elimination, and reduction [13] (Scheme 2). The epoxidation and epoxide opening by base conducts to the hydroxyderivative **12**, which can be eliminated by protonation with *p*-toluensulfonic acid (TsOH) to afford **13**. The reduction of cyano group with diisobutyl aluminium hydride (DIBAL-H) results in safranal **2**.

**Scheme 3:**
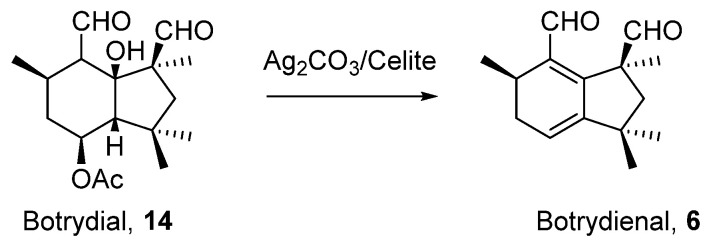
Transformation of botrydial **14** into botrydienal **6** by reaction with Ag_2_CO_3_.

The transformation of botrydial **14** into its derivative botrydienal **6** (Scheme 3) can be triggered under Fetizon conditions with a quantitative yield, as described by Durán-Patrón et al. [14].

**Scheme 4:**
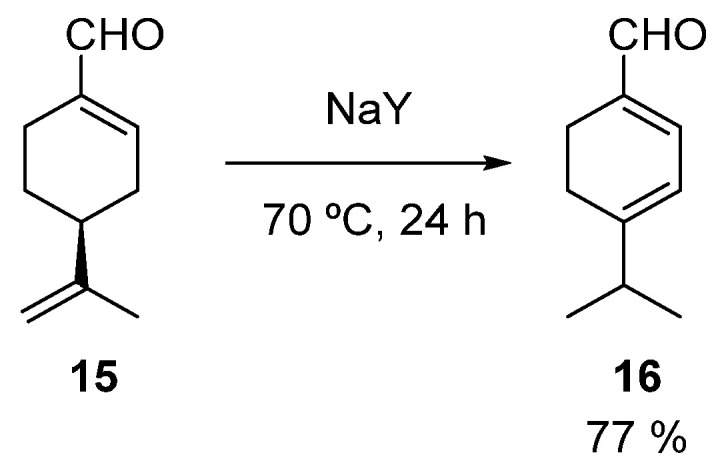
Isomerization of dienal **15**.

Starting from α,β,ε,ζ-unconjugated dienals such as **15**, the unconjugated double bond can be isomerized to form the corresponding α,β,γ,δ-unsaturated aldehyde (**16**) using zeolite-NaY in dry conditions (Scheme 4). Longer reaction periods conduct the aromatization products, although this can be easily controlled [15].

### 2.2. Oxidation

**Scheme 5:**
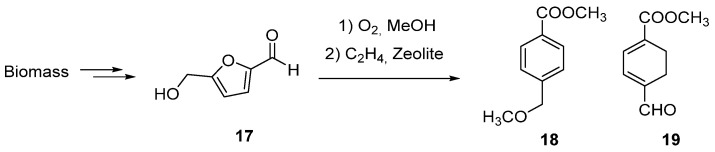
Synthesis of terephthalic derivatives from biomass.

Using the furfural derivative **17** from a renewable source for its conversion into terephthalic acid, one can obtain the intermediate **18** and the dienic side-product **19** (Scheme 5). The transformation takes place in two steps. Firstly, oxidation and then C_2_H_4_ addition catalyzed by silica molecular sieves showing zeolite-β topology containing Lewis acid centers (Zr-β or Sn-β). Despite the obtention of **19** being possible by using both catalysts, when Sn-β is used instead of Zr-β, **19** is obtained with higher yields [16,17]. Low molecular weight 1,3-cyclohexadienals have special relevance in perfumery, with some terpene derivatives being of interest, such as safranal **2** and analogues. In this regard, the conversion of thujone (waste product in forest industry) into different derivatives has been studied, obtaining safranal **2** and analogues [18].

### 2.3. Vilsmeier–Haack Formylation

**Scheme 6:**
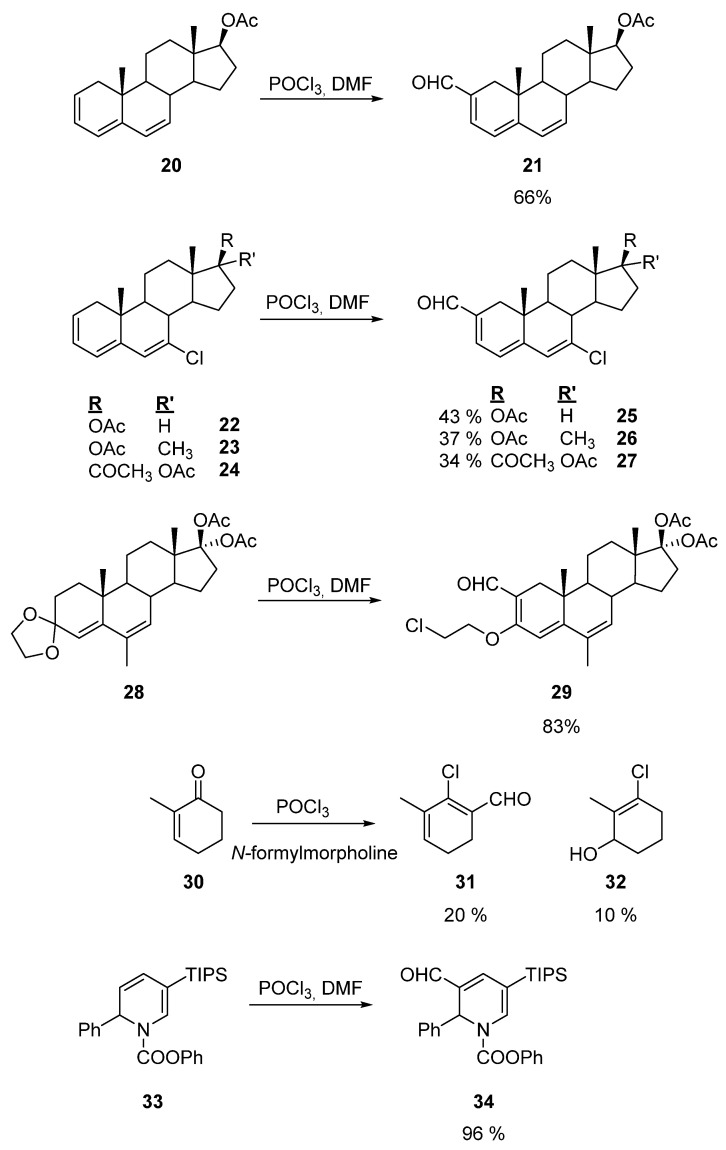
Synthesis of cyclohexandienals under Vilsmeier–Haack reaction conditions.

When the reactant presents the appropriate enic system, the Vilsmeier reaction can afford the 1,3-cyclohexadien-1-al scaffold. Among several examples, it can be found that the application to tetracyclic triterpenic derivatives **20**, **22**–**24**, and **28** [19,20,21,22,23] can be transformed into their corresponding derivatives **21, 25**–**27**, and **29** with moderate or good yields (Scheme 6). Equivalent conditions with POCl_3_ in the presence of *N*-formylmorpholine as formylating agent can be used. Such is the case of the transformation of **30** to dienal **31**, although lower yield is attained since hydroxyderivative **32** is detected as a by-product [24]. Heterocyclic compounds such as **33** can be transformed into the corresponding heterocyclohexa-1,3-dienals (**34**) under Vilsmeier–Haack conditions (Scheme 6) [25].

**Scheme 7:**
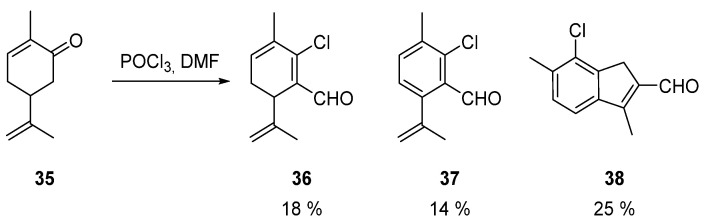
Carvone **35** formylation under Vilsmeier–Haack conditions.

Vilsmeier reaction conditions applied to carvone **35** affords the corresponding 2-chloro-1,3-cyclohexadienal derivative **36** (Scheme 7). However, these reaction conditions show poor yield in the obtention of this product, since two by-products (**37** and **38**) are generated with significant yields.

**Scheme 8:**
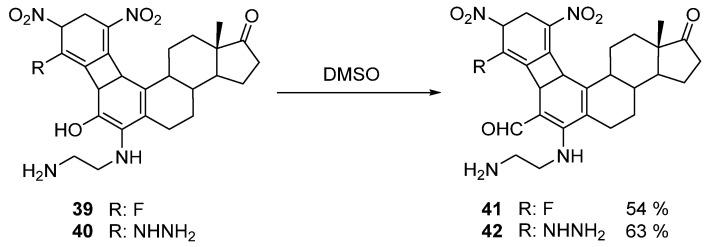
Formylation reaction in the synthetic preparation of cyclohexadienal motif.

Other formylating agents such as DMSO can also be used, starting from the corresponding conjugated enol (Scheme 8). The 1,3-cyclohexadienal obtained has been used as a precursor of diazocine steroid derivatives through eight-member ring formation [26]. Such a transformation is shown below in the reactivity section.

### 2.4. Aldol-Type Reactions and Horner Olefinations

**Scheme 9:**
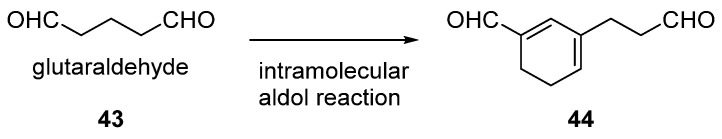
Self-dimerization of glutaraldehyde **43**.

The 1,3-cyclohexadienal scaffold can also be obtained as a result of glutaraldehyde self-assembly via intramolecular aldol reaction polimerization, as shown in Scheme 9 [27,28]. Through this intramolecular aldol reaction, 1,3-cyclohexadienal compound substituted in C-3 can be obtained.

**Scheme 10:**
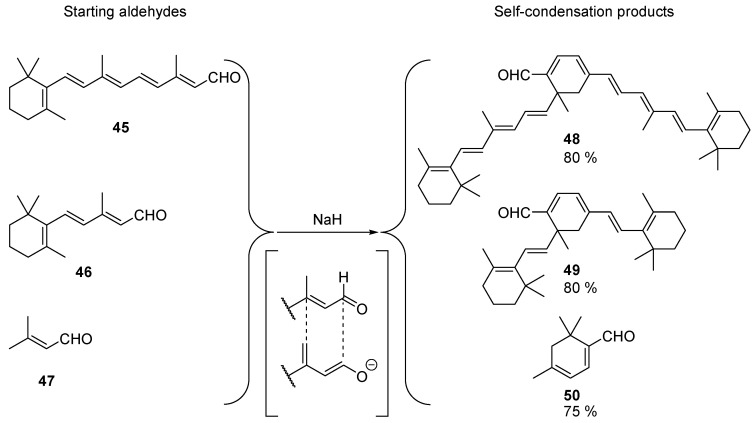
Self-condensation of α,β-aldehydes in the formation of 1,3-cyclohexadien-1-al units.

Aldol reaction and condensation of α,β-aldehydes can be performed in self-condensation or cross-condensation modality.The self-condensation of all-*E*-retinal **45** can be performed in the presence of sodium hydride (Scheme 10) [29]. The enolate intermediate reacts with the starting aldehyde and cyclizes, forming the 1,3-cyclohexadien-1-al scaffold **48**. In the enolate intermediate, the β-methyl group holds the negative charge and reacts at the conjugated position of the aldehyde in a process where the *Z*/*E* configuration of double bound is not determinant, allowing in any case the condensation and formation of dienal.

**Scheme 11:**
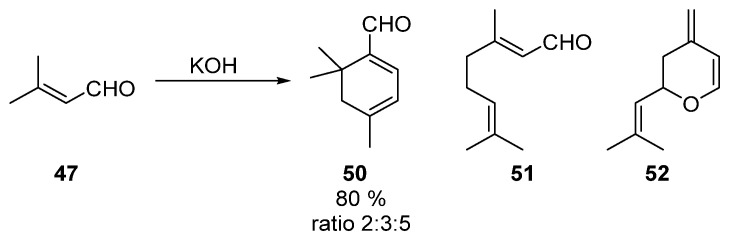
Self-condensation of senecialdehyde **47** in the formation of 1,3-cyclohexadien-1-al **50**.

Similar results are obtained with senecialdehyde **47**, self-condensation reaction promoted by alkali [30]. In this case, a global yield of 80% is obtained, and compounds **50–52** are obtained in a ratio 2:3:5 (Scheme 11). Cyclodimerization also occurs when crotonaldehyde is treated with solid base catalysts of metal oxides and silica. In this case, the obtained diene can evolve by dehydrogenation and aromatization [31]. 

**Scheme 12:**
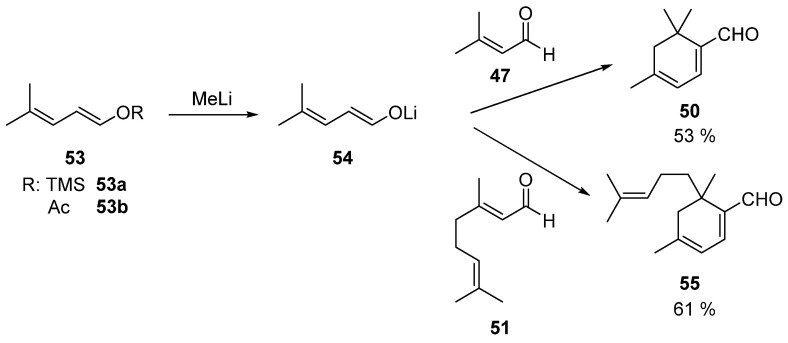
Cross-condensation of unsaturated aldehydes in the formation of 1,3-cyclohexadien-1-als.

Trimethylsilyl and acetate enolderivatives **53** serve as starting material for obtaining 1,3-cyclohexadien-1-als through the lithium enolate formation (Scheme 12). Intermediate **54** can be accessed via metalation of **53** with MeLi. The subsequent addition of a conjugated aldehyde **47** or **51** yields 1,3-cyclohexadienals **50** or **55**, respectively. In this manner, lithium enolates are a versatile tool in organic synthesis, allowing for the cross-condensation between two different unsaturated aldehydes, opening the possibilities among the non-chiral condensation methodology [32].

**Scheme 13:**
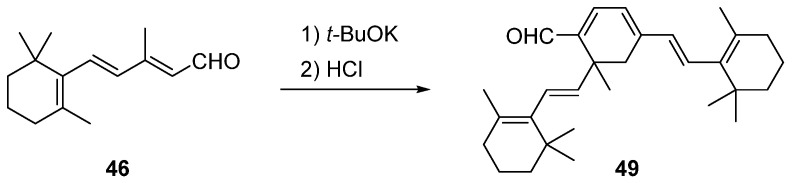
Self-condensation of α,β-unsaturated aldehyde **46** in the formation of 1,3-cyclohexadien-1-al **49**.

The aldol reaction between aldehyde **46** and its enolate formed in presence of potassium *tert*-butoxide (*t*-BuOK) leads to the dimeric intermediate that in acidic medium evolves to form the self-condensation product **49** (Scheme 13) [33]. α,β-Unsaturated nitriles produce analogue condensation mechanism, giving the corresponding 2-amino-1,3-cyclohexadien-1-carbonitrile scaffold; these derivatives can be easily converted to their corresponding aldehydes.

**Scheme 14:**
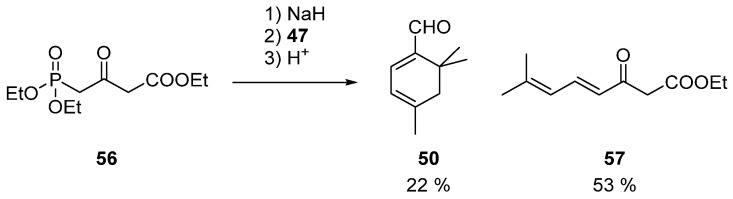
Michael and Horner–Wadsworth–Emmons reactions in the synthesis of cyclohexadienal **50.**

Studying the reactivity of ethyl 4-(diethoxyphosphinyl)-3-oxobutanoate **56** with enals (Scheme 14), one finds that after conjugated Michael addition to the corresponding enal and subsequent Horner reaction over the free carbonyl compound, the formation of cyclic compounds such as dienal **50** (22%) and the major product, the direct Horner reaction product **57** (53%), occurs [34]. The strong basic medium allows for the self-condensation of crotonaldehyde [35].

**Scheme 15:**
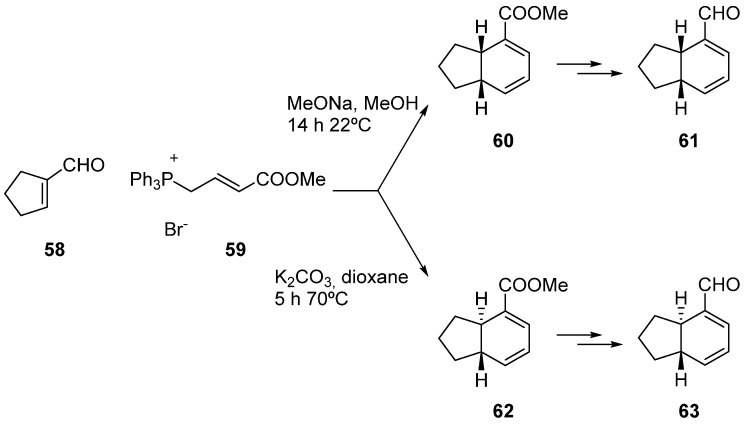

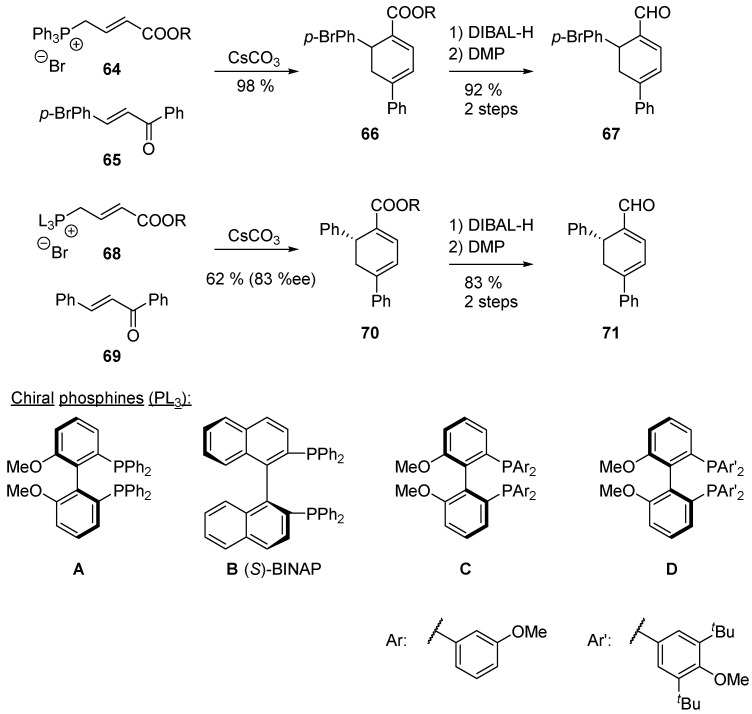
Michael and Horner–Wadsworth–Emmons reactions in the synthesis of cyclohexadienals.

Starting from linear starting materials, the cyclization to form six-membered rings can be performed in two steps through Horner olefination of α,β-unsaturated aldehydes and Michael addition to the resulting unsaturated system (Scheme 15). Ester reduction yields the 1,3-cyclohexadienal scaffold, wherein relative configuration can be controlled by using different bases for the conjugated addition. This methodology has been used in the total synthesis of natural products containing 1,3-cyclohexadien-1-al scaffolds such as (±)-*cis*/*trans*-2,3,3a,7a-tetrahydro-1*H*-indene-4-carbaldehyde **61** and **63** that have been prepared starting from aldehyde **58** and the ylide of **59** [6]. Similar methodology has been applied for obtaining 1,3-cyclohexadienals due to their interest as intermediates of polysubstituted aromatic compounds, as indicated in the preparation of **67** from **64** and **65** (Scheme 15) [36]. The use of chiral phosphines in this reaction opens the possibility of asymmetric synthesis for this scaffold, as exemplified in the preparation of **71** from **68** using phosphine derivative **C**, obtaining good enantiomeric excess (ee) and moderate yield [16].

### 2.5. Pericyclic Reactions: Diels–Alder and Electrocyclizations

**Scheme 16:**
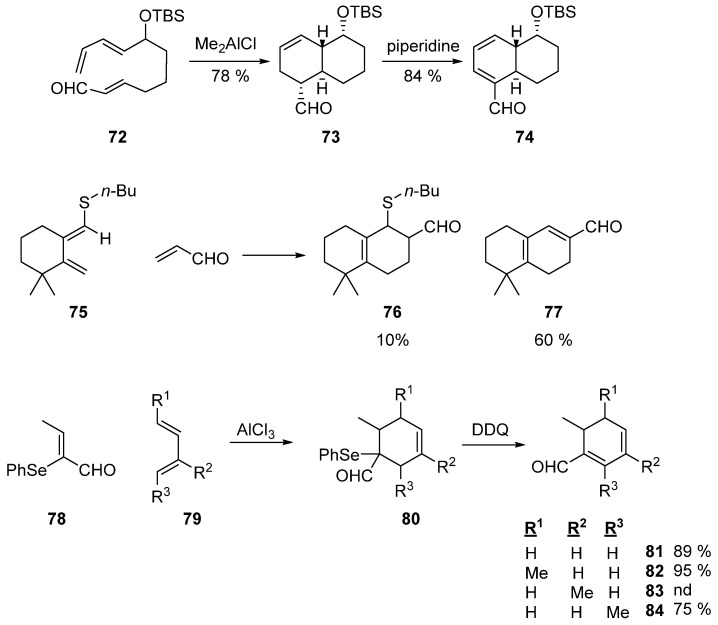
Diels–Alder reactions towards the synthesis of 1,3-cyclohexadien-1-al scaffold.

The Diels–Alder reaction is the most important and versatile reaction in synthetic organic chemistry for preparing six-membered rings. The 1,3-cyclohexadien-1-al scaffold can be afforded via Diels–Alder reaction by cycloaddition followed by base-promoted β-elimination. For example, intramolecular Diels–Alder reaction of **72** catalysed by a Lewis acid produces the adduct intermediate **73**, which can be transformed in the corresponding dienal **74** by elimination reaction (Scheme 16) [37]. Other alternatives for the obtention of 1,3-cyclohexadienal after a Diels–Alder reaction consist of the presence of a good leaving group positioned on the allylic position of the reaction adduct. Starting from the alkylthioenolate **75** [38], by treatment with acrolein, the adduct **76** is formed in low yield, resulting as major product the dienal **77** (60% yield), which could be obtained in higher yield by augmenting the reaction time. When the Diels–Alder reaction is performed with 2-(4-chlorophenylseleno)-2-butenal **78** as dienophile [39], the oxidative deselenylation (with 2,3-dichloro-5,6-dicyano-1,4-benzoquinone (DDQ)) of intermediate **80** conducts to the desired 1,3-cyclohexadienal (**81**–**84**) with excellent yields (Scheme 16). However, this oxidation must be carefully controlled, as DDQ can aromatize the ring obtaining the benzaldehyde derivative.

**Scheme 17:**
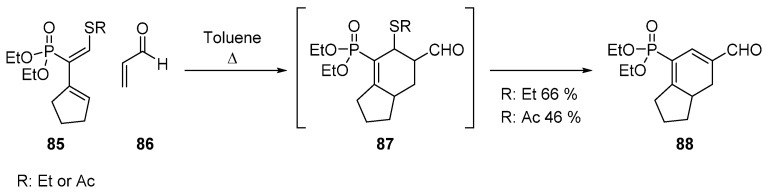
Diels–Alder reaction towards the synthesis of 1,3-cyclohexadien-1-al scaffold.

Another example of Diels–Alder cycloaddition wherein a high regioselectivity is reached thanks to the sulfur-containing diene is shown in Scheme 17 [40,41]. The sulfide acts as a regioselective assistant as well as a good leaving group once the adduct is formed, yielding the 1,3-cyclohexadienal. The reaction of the diene **85** with acroleine **86** yields the typical mono-olefin adduct of the Diels–Alder reaction that after elimination of allylic ethylsulfide group yields 1,3-cyclohexadienal **88**. The reaction can be catalyzed by ZnBr_2_, allowing a lower activation energy and thus heating at 60 °C instead of 100 °C. The reaction can be even carried out at 20 °C when LiClO_4_/Et_2_O is added as catalyst, but higher reaction times are needed.

**Scheme 18:**
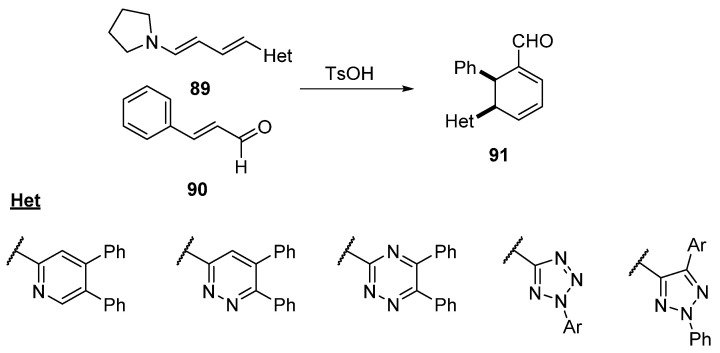
Diels–Alder reaction towards the synthesis of 1,3-cyclohexadien-1-al scaffold.

The Lewis acid- or Brønsted acid-catalyzed Diels–Alder reaction between cinnamaldehyde **90** and different enamines **89** resulted in 1,3-cyclohexadienal substituted in 5 and 6 positions (Scheme 18). Among the Lewis and Brønsted acids used, TsOH, boron trifluoride ethyl etherate BF_3_·OEt_2_, LiClO_4_, TiCl_4_, and catalytic TsOH (0.2 equivalents) proved most efficient [42].

**Scheme 19:**
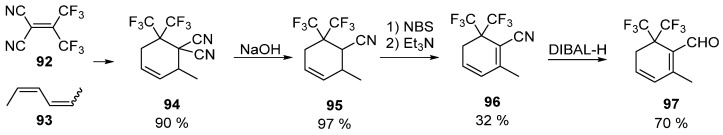
Diels–Alder reaction towards the synthesis of hexafluorinated safranal **97**.

By using the Diels–Alder reaction to form the 1,3-cyclohexadienal, one can employ other starting materials such as 2,2-bis(trifluoromethyl)-ethylene-1,1-dicarbonitrile **92,** a very strong dienophile (Scheme 19) [43]. However, three transformation steps are needed to form the 1,3-cyclohexadienal from adduct **94**. The first step, the Diels–Alder reaction between the dicarbonitrile **92** and the diene **93** occurs in an excellent yield (90%) and can be improved up to 97% when **93** is in excess and increasing reaction time. The elimination of a nitrile group in **94** is carried out by reaction with NaOH in aqueous ethanol, resulting in **95** (97% yield). The formation of diene **96** from **95** takes place in two steps: allylic bromination with *N*-bromosuccinimide (NBS) and elimination reaction with triethylamine (Et_3_N). The bromination is a critical step wherein monobromination must be controlled, showing moderate conversion over these two steps (63%) and low yield (32%). Finally, reduction of nitrile **96** with DIBAL-H gives bis-trifluoromethyl-1,3-cyclohexadienal **97** in moderate yield. 

**Scheme 20:**
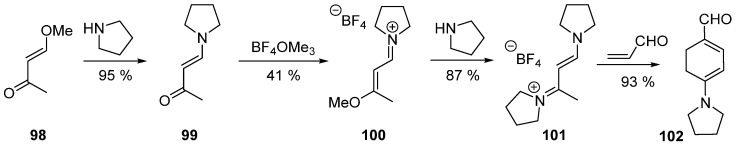
Diels–Alder reaction of vinamidium salts and unsaturated aldehydes.

Other dienes derived from vinamidinium salts are versatile intermediates to perform Diels–Alder reactions (Scheme 20) [44]. For example, starting from the previously prepared vinamidinium salt **101**, reaction with acrylaldehyde results in cyclohexadiene **102**. These vinamidinium salt diene precursors are prepared in three steps, starting from unsaturated methylketones such as **98** by reaction with a secondary amine, pyrrolidine in this case. The resulting enamine **99** is tautomerized to the iminium salt **94**, and then a second equivalent of secondary amine is added to form the vinamidinium salt **101**.

**Scheme 21:**
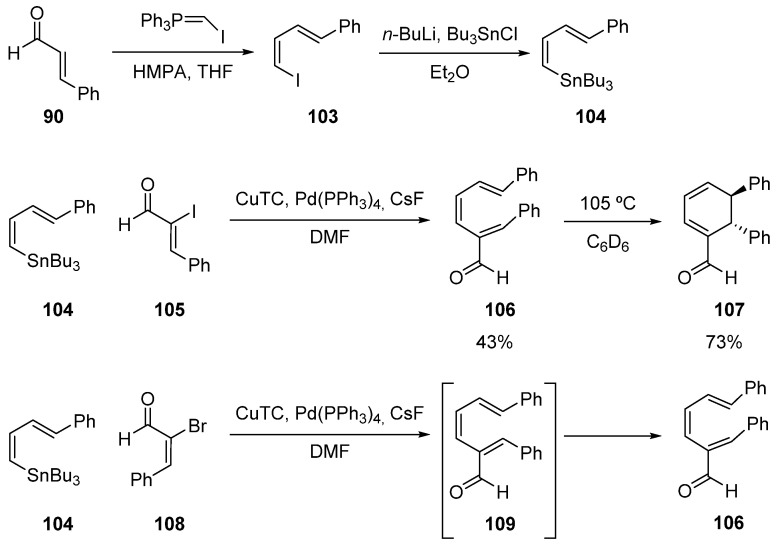
Synthesis and thermal cyclization of hexatrienes **106** and **109**; CuTC = copper(I) thiophene-2-carboxylate.

Starting from linear trienes, Lewis acid-catalyzed electrocyclizations form the 1,3-cyclohexadiene-1-carbaldehyde fragment (Scheme 21) [45]. The *Z*/*E* configuration of double bonds as well as the presence of large substituents in 5 position are key points in terms of establishing the energy barrier for the electrocyclization to occur.

**Scheme 22:**
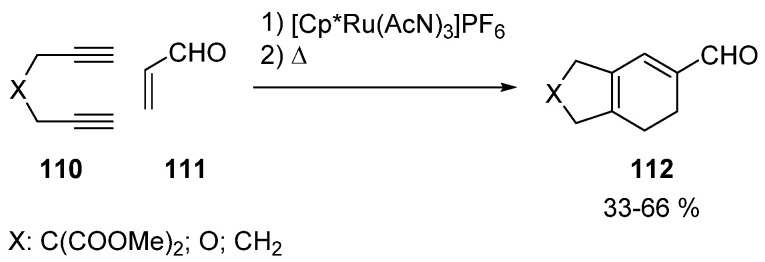
Ruthenium catalyzed electrocyclization in the preparation of 1,3-cyclohexadienal scaffold.

Organoruthenium complexes can yield 1,3-cyclohexadienal scaffold through a 6e-π electrocyclization (Scheme 22). The reaction takes place between a 1,6-diyne **110** and acroleine [46].

### 2.6. Organometallic Synthetic Procedures

**Scheme 23:**
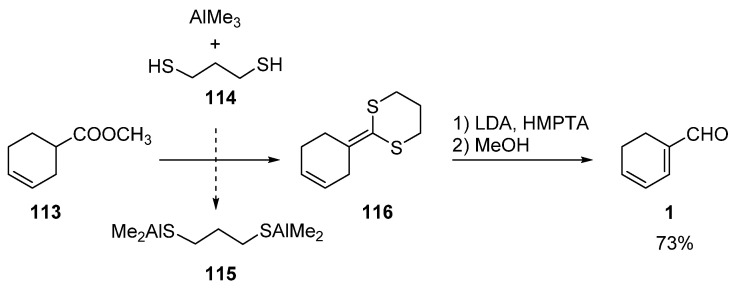
Employment of organoaluminium reagent in the synthesis of cyclohexadienal **1**.

According to the reaction conditions reported by Corey et al. [47], γ,δ-unsaturated esters can be used as precursors of 1,3-cyclohexadien-1-als (Scheme 23). The transformation of **113** into intermediate **116** by reaction with the previously prepared bis(dimethylaluminium)-1,3-propanethiol followed by a two-step reaction of lithium diisopropylamide/hexamethylphosphoramide (LDA/HMPTA) anion formation and addition of methanol for quenching leads to the desired product **1** in good yield.

**Scheme 24:**
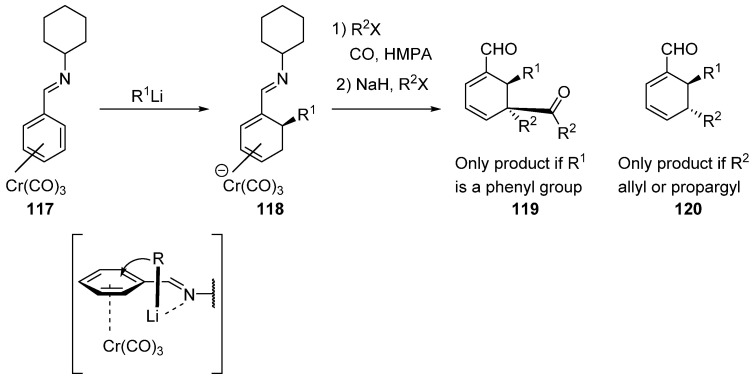
Cr(CO)_3_ complex-mediated dearomatization in the synthesis of cyclohexadienals.

An organometallic method using Cr(CO)_3_ complex and benzaldehyde consisting of its dearomatization and chemoselective nucleophile addition has been reported (Scheme 24) [48]. Starting from benzaldehyde, preparation of the corresponding phenylmethanimine–tricarbonylchromium complex **117** is performed. Addition of a nucleophile is followed in the formation of anionic cyclohexadienyl complex **118** that is trapped with different bromides, providing at the end the 1,3-cyclohexadienal substituted in 5 and 6 positions. Different ratio of products **119** and **120** is obtained depending on the R^1^ and R^2^ groups. Compound **119** is preferentially formed when R^1^ is a phenyl group, while cyclohexadienal **120** is formed selectively when R^2^ is an allyl or propargyl group.

**Scheme 25:**
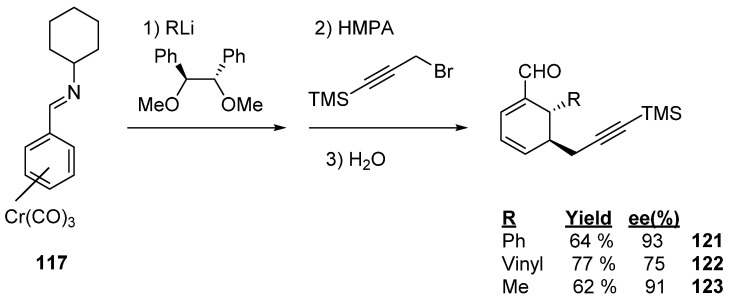
Cr(CO)_3_ complex-mediated dearomatization in the presence of chiral ligands.

In these complexes, **117**, the imine group, acts as an auxiliary that directs R^1^ to the ortho position via lithium–nitrogen interaction (Scheme 25). External chiral ligands can be added to the reaction in order to induce stereoselectivity in the product [49], showing the highest enantioselectivities when PhLi is used as nucleophile. The external ligand forms an oligomeric entity with the nucleophile acting as a bridge instead of a simple chelating process.

**Scheme 26:**
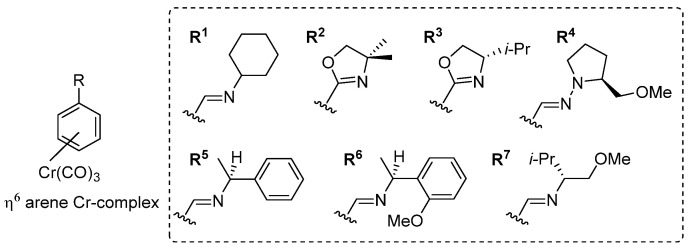
Selection of Cr(CO)_3_ complexes used in dearomatization reactions for the preparation of cyclohexadienals.

A selection of nitrogen-containing precursors that can be used to form the corresponding tricarbonylchromium complex is shown in Scheme 26 [48,49,50,51]. These precursors can be prepared using the corresponding amine (R-NH_2_) and the benzaldehyde unit. When the nitrogen-containing precursor is optically pure, the reaction shows high diastereoselectivity in the addition of nucleophiles in an asymmetric manner (up to 98% diatereomeric excess, de), as in the case of R^4^.

**Scheme 27:**
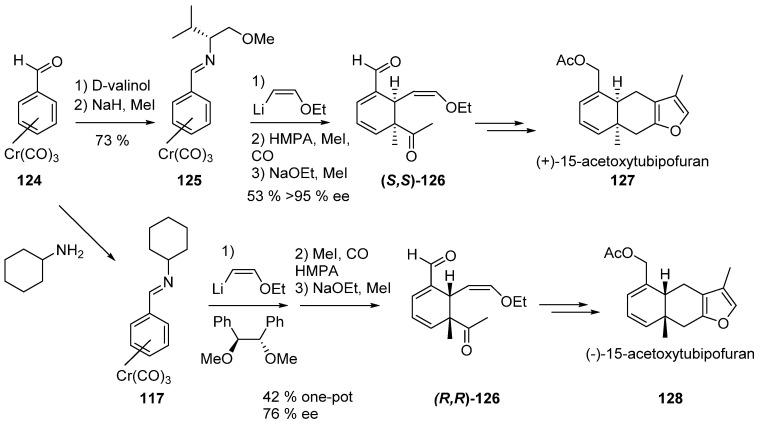
Cr(CO)_3_ complex-mediated dearomatization in the synthesis of cyclohexadienals towards acetoxytubipofuran.

The tricarbonylchromium-mediated dearomatization is a useful tool to access substituted cyclohexadienals. It has been used to synthesize acetoxytubipofuran, as a reacemic mixture [52] and also as the two separated enantiomers [51]. The natural product, isolated from *Tubipora musica,* is an eudesmane marine sesquiterpene that has been synthesized from dienal (*S*,*S*)-**126** and (*R*,*R*)-**126** as key intermediates, which in turn have been prepared according to the synthetic pathway described in Scheme 27. 

### 2.7. Organocatalytic Methodologies

**Scheme 28:**
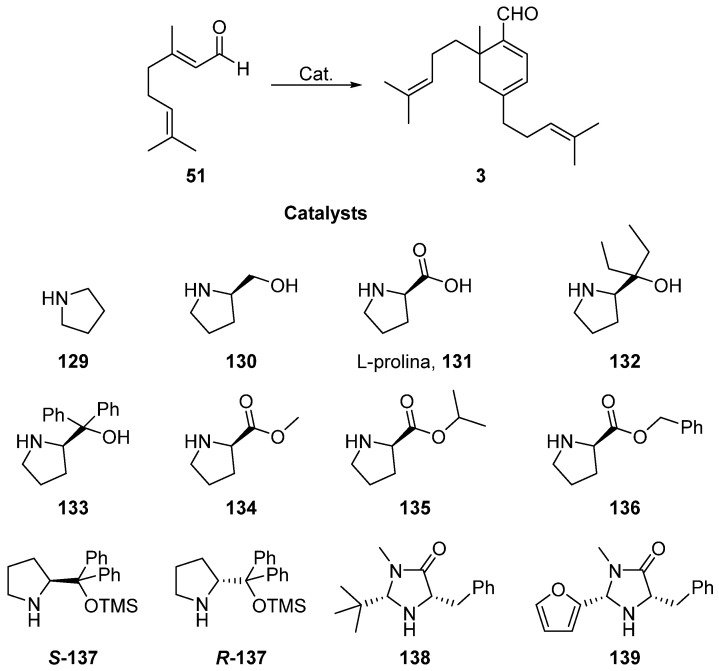
Organocatalyzed synthesis of 1,3-cyclohexadien-1-als from citral **51**.

The access to chiral 1,3-cyclohexadienals can be accomplished by using organocatalysis. This methodology is a versatile method that uses as starting materials an α,β-unsaturated aldehyde and a α,β-unsaturated aldehyde with a methyl or methylene group in β. A high number of proline derivatives have been used as organocatalysts (**129**–**139**), as shown in Scheme 28 [53]. Two different mechanisms have been proposed: (4 + 2) cycloaddition or Michael addition and cyclization [53].

**Scheme 29:**
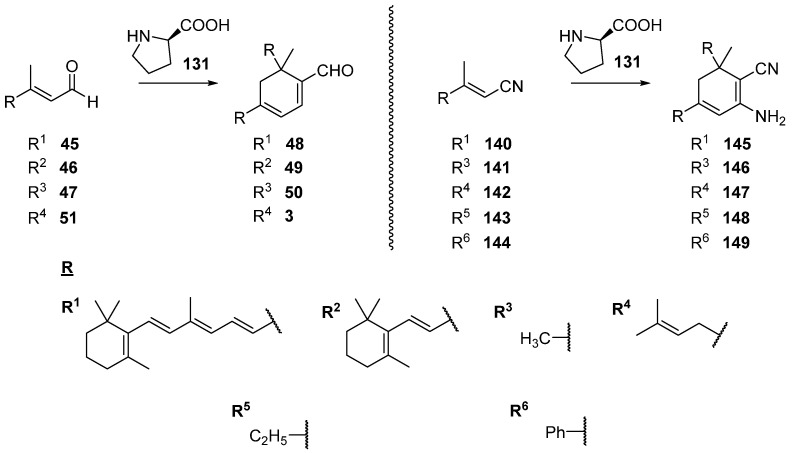
Organocatalytic self-condensation of α,β-unsaturated aldehydes towards 1,3-cyclohexadien-1-al compounds.

The first use of proline as a catalyst begins with its application in the biomimetic cyclization of citral, synthesizing unsaturated aldehydes (Scheme 29) [54,55]. However, the specific use of proline to synthesize 1,3-cyclohexadienals occurred in 1992 [56], where the reaction of this aminoacid with different α,β-unsaturated aldehydes promoted their self-condensation. The good enantiomeric excess (up to 65%) resulted in a deepening of the research on the use of proline as organocatalyst as well as the development of proline analogues with improved catalytic properties. L-proline is used in excess in these reactions and the self-condensation product is always detected. When other proline derivatives such as 4-hydroxy-L-proline, D-proline, and *cis*-4-hydroxy-D-proline are used, the self-assembly of the aldehydes is observed; however, this self-condensation product of citral is not observed when Trp, His, Arg, Gly, or ILeu are used as catalysts for citral dimerization.

This condensation takes place through Schiff-base formation as a synthetic intermediate that reacts with other equivalents of α,β-unsaturated aldehyde yielding the 1,3-cyclohexadien-1-al core. However, sometimes the intermediate Schiff-base is stable enough and can be isolated, as in the case of the corresponding Schiff-base of L-proline and retinal [53].

In this sense, even when cross-condensation is performed, the autocondensation reaction should be considered as a possible background reaction. In the case of the use of citral, there is frequently the presence of the citral homodimer among the reaction products [57,58], as well as in organocatalyzed cycloaddition in multicomponent reactions [59].

**Scheme 30:**
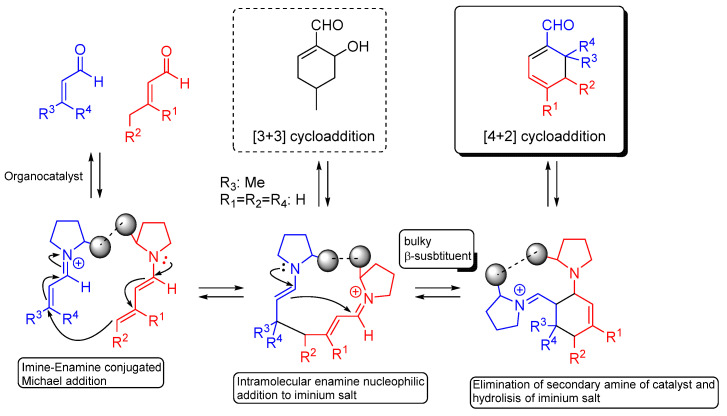
Organocatalyst-mediated cycloaddition of α,β-unsaturated aldehydes towards 1,3-cyclohexadien-1-al compounds.

Among the different organocatalysts, the aminocatalysts proline, MacMillan’s **138** or **139**, and Hayashi-Jorgensen’s **137** [60] catalysts can promote Michael addition by iminium salt formation with the acceptor enal. On the other hand, a carbonyl donor can be activated by forming the enamine by addition of the organocatalyst and ensuing Michael addition. Thus, iminium salt-enamine formation turns out to be key in these organocatalytic pathways (Scheme 30). Aldehydes such as crotonaldehyde reacts with L-proline through a [3 + 3] cycloaddition, but when other α,β-unsaturated aldehydes with β-substituents react in the same conditions, the observed product is the (4 + 2) adduct [61]. The mechanistic explanation suggests that increasing the steric hindrance in β-position of these series of aldehydes eases the indirect Mannich reaction pathway and (4 + 2) adducts instead of the [3 + 3] cycloaddition products when carbon 3 is more accessible.

The reaction of different aldehydes in the presence of L-proline yields the corresponding (4 + 2) adducts, and results are shown in Table 1 [61]. Pyrrolidine can also be a good catalyst for the (4 + 2) cycloaddition [62], where a wide scope is given. Subsequent aromatization of the diene adducts by reaction with DDQ or MnO_2_ gives the benzaldehyde derivatives, providing, in this way, a good methodology for controlled substitution of benzaldehyde derivatives.

**Scheme 31:**
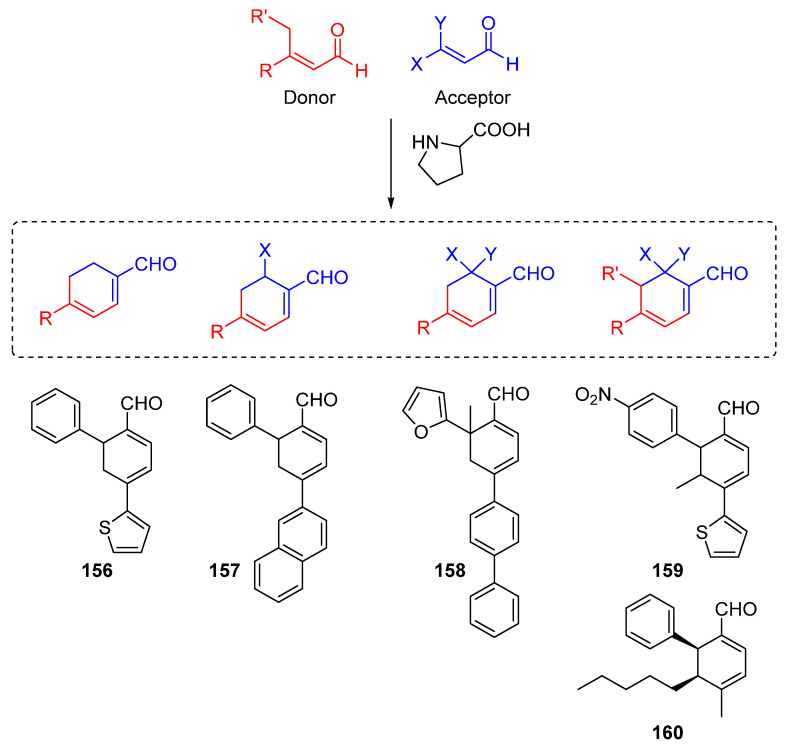
Examples of cyclohexadienals obtained in L-proline-mediated reaction of α,β-unsaturated aldehydes.

Watanabe et al. synthesized a high number of 1,3-cyclohexadienal derivatives with different substitution degrees using proline as organocatalyst (Scheme 31) [63]. By this procedure, the homodimerization product can be observed in a variety of yields (5 to 75%), and moderate ee (36 to 72%) are observed, depending on the combination of donor–acceptor aldehydes.

The optimization of the reaction consists of pre-forming the iminium salt of donor aldehyde-proline and then addition of acceptor aldehyde, minimizing in this way the formation of the homo-dimmer.

**Scheme 32:**
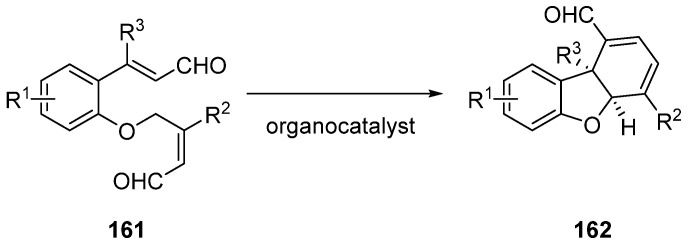
Intramolecular organocatalytic formation of cyclohexadienal unit.

Intramolecular organocatalyzed cycloaddition has been screened in a variety of cinnamaldehyde derivatives, obtaining dihydrodibenzofurans with good enantioselectivity and yields (Scheme 32) [64].

**Scheme 33:**
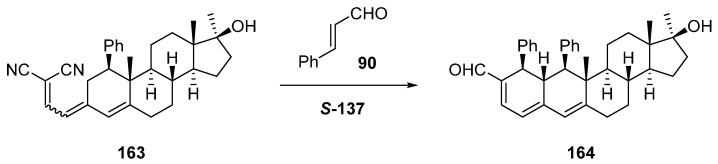
Organocatalyst-mediated cycloaddition for the synthesis of cyclohexadienal core.

A formal eliminative (4 + 2) cycloaddition can be performed, starting from a γ-enolizable α,β-unsaturated carbonyl compound (diene) and an enal (dienophile) (Scheme 33) [65]. The addition of an organocatalyst allows for a diastereoselection in the cycloaddition through the formation of the Schiff-base. After screening the reaction conditions and demonstrating the general applicability of the (4 + 2) eliminative cycloaddition, one can apply the procedure to steroid-based dicyano substrates.

**Scheme 34:**
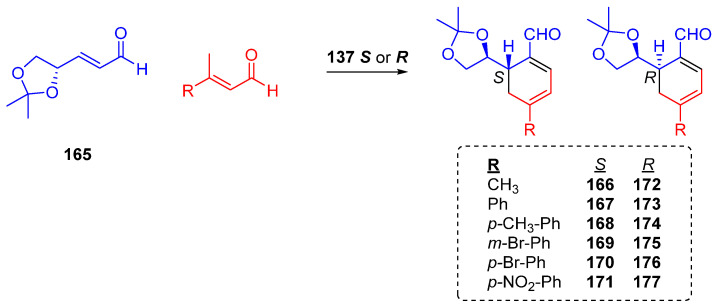
Organocatalytic-mediated synthesis of chiral 1,3-cyclohexadienals.

The same methodology has been applied to the synthesis of chiral 1,3-cyclohexadienals, where a high diastereoselection has been reported, showing up to a 85:15 diastereomeric ratio and moderate to good yield (Scheme 34) [66].

**Scheme 35:**
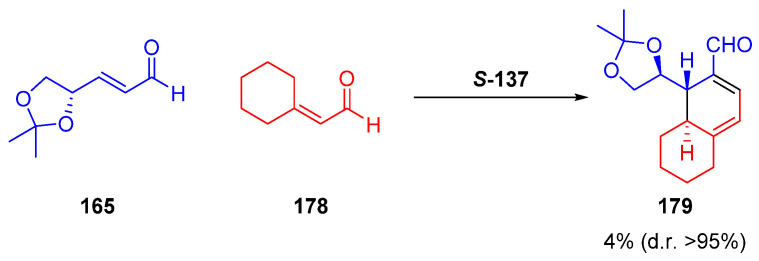
Organocatalytic-mediated synthesis of chiral 1,3-cyclohexadienal **179**.

It is important to highlight the relevance of the β-substitution in donor aldehyde, when instead of a methyl group, the conjugated unsaturation is an exocyclic double bond, and the reactivity for the cyclohexadiene formation decreases, significantly reducing the reaction yield. However, in this case, two chiral centers are generated in the reaction product with excellent diastereoselectivity (Scheme 35).

**Scheme 36:**
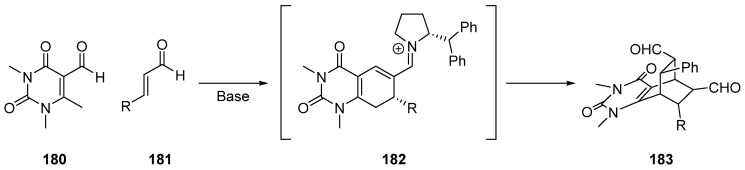
Organocatalytic-mediated synthesis of cyclodienal and subsequent reaction with conjugated aldehyde **181**.

This organocatalytic process is a very useful tool that can be applied to one-pot reactions where successive cycloadditions are produced. Saktura et al. reported this reaction cascade of uracil and conjugated aldehydes where the 1,3-cyclohexadienal is synthesized in situ as the synthetic equivalent, the enamine **182** (Scheme 36) [67]. The enamine **182** in basic media reacts again with the other equivalent of the conjugated aldehyde **181** to form bicyclo [2.2.2]octane **183**.

**Scheme 37:**
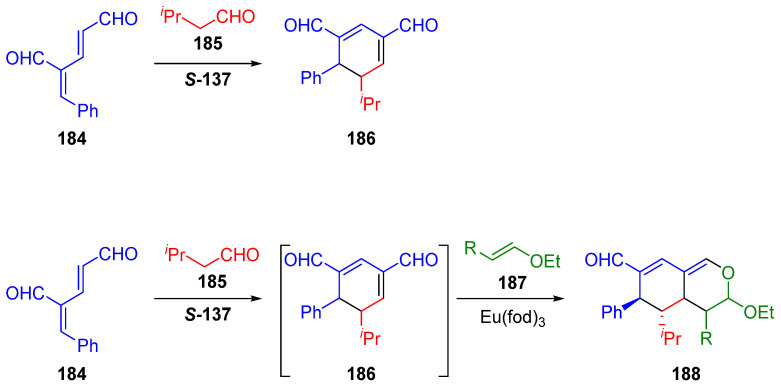
Synthesis of cyclohexadienal compounds and subsequent cycloaddition reaction.

Other examples of 1,3-cyclohexadien-1-als obtained as synthetic intermediates can be found in the work of Jorgensen, who synthesized tetrahydroisochromenes starting from oxadendralenes (Scheme 37) [68]. In the aim of obtaining the tetrahydroisochromene derivatives in a three-component reaction, research first accomplished screening the reaction conditions to obtain dienal **186**. Good yield and diastereoselectivity is obtained in the reaction of aldehyde **184** and **185** in the presence of organocatalyst ***S*-137**, obtaining the oxadendralene intermediate (Scheme 37). Once this reaction is viable, the next step involves the reaction with vinyl ether **187**. The cyclization occurs in the presence of a co-catalyst such as Eu(fod)_3_ (10 mol %), resulting in the desired tetrahydrochromene **188**.

## 3. Reactivity of 1,3-Cyclohexadien-1-Als

The 1,3-cyclohexadien-1-carboxaldehyde unit is a highly functionalized small molecule with high potential as building block, and as such it has been used widely for the construction of a great variety of compounds. Reactivity of the functional groups in the scaffold has been harnessed, and a great deal of transformations involve typical reactions of the double bond functional group, aldehyde functional group, and the α,β-unsaturated aldehyde system. Several examples of each of these three types of reactivity are shown below.

### 3.1. Reactions Involving Double Bond(s)

**Scheme 38:**
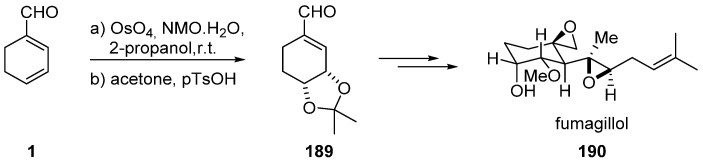
Dihydroxilation of 1,3-cyclohexadien-1-carboxaldehyde in the total synthesis of fumagillol **190**.

1,3-Cyclohexadien-1-carboxaldehyde **1** was considered as a starting material in the total synthesis of fumagillol **190** [69,70], in which the first step implies dihydroxylation of the more electron rich γ,δ-double bond (Scheme 38).

**Scheme 39:**
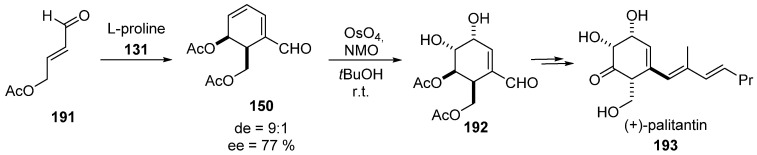
Dihydroxilation of cyclohexadienal **150** in the total synthesis of (+)-palitantin **193**.

Dihydroxylation reaction of cyclohexadienal **150** (Scheme 39) to diol **192** was also considered in the total synthesis of (+) palitantin [71], a natural compound with antifungal [72] and antibiotic activities [73]. The cyclohexadiene carboxaldehyde intermediate **150** was obtained by self-dimerizacion of (*E*)-4-acetoxycrotonaldehyde **191** in the presence of L-proline [71].

**Scheme 40:**
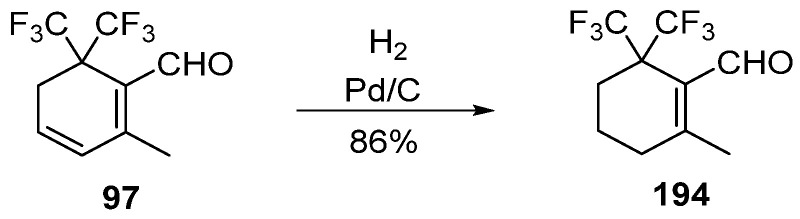
Hydrogenation of hexafluorinated safranal **97**.

The preparation of hexafluorinated safranal **97** has been achieved by Haas et al. (Scheme 40) [43]. Hydrogenation of the C3–C4 double bond in such a compound in the presence of palladium on charcoal catalyst led to another hexafluorinated natural product, 7,7,7,8,8,8-hexafluoro-β-cyclocitral **194**.

**Scheme 41:**
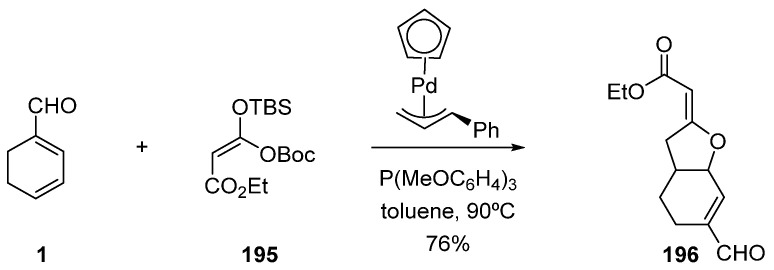
Oxyallyl cycloaddition reaction with cyclohexadienal.

Trost et al. recently presented an interesting study about the catalytic palladium oxyally cycloaddition reaction with conjugated dienes [74], wherein 1,3-cyclohexadiene-1-carboxaldehyde was also tested, giving the corresponding bicyclic tetrahydrofuran product **196** with extraordinary chemoselectivity (Scheme 41). This shows once more the versatility of the cyclohexadienal unit, which can be involved in many different transformations.

**Scheme 42:**
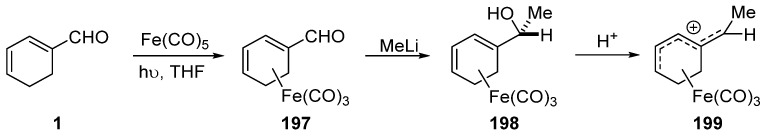
Formation of tricarbonyl iron complex of 1,3-cyclohexadiene-1-carboxaldehyde **1**.

Tricarbonyl iron complex of 1,3-cyclohexadiene-1-carboxaldehyde **1** has been prepared in a photochemical reaction with iron pentacarbonyl (Scheme 42) [75]. Such complex **197** was synthesized in the route to structurally constrained tricarbonyl (*trans*-pentadienyl) iron cations **199** to study acid catalyzed rearrangements.

**Scheme 43:**
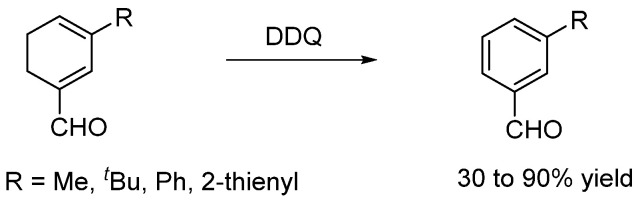
Aromatization reaction of cyclohexadienals.

Aromatization of cyclohexadienals has been used to prepare aromatic aldehydes (Scheme 43) [76]. Oxidation with 2,3-dichloro-5,6-dicyano-1,4-benzoquinone (DDQ) allows for the aromatization reaction with moderate to high yields.

**Scheme 44:**
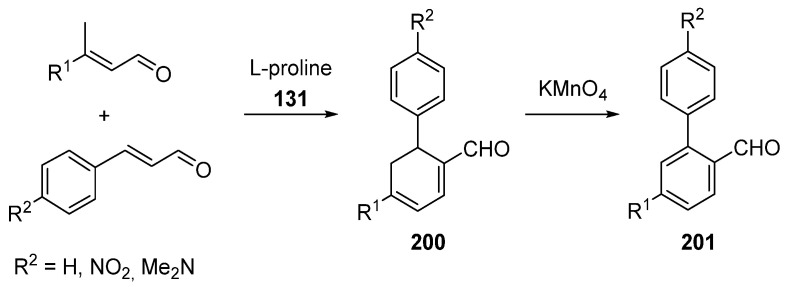
Aromatization reaction of phenyl-substituted cyclohexadienals.

Phenyl-substituted cyclohexadienals **200** were found to spontaneously aromatize in low conversion of 5–15% in a period of one to several days [63]. However, aromatization could also be performed in quantitative yields by reaction with potassium permanganate (Scheme 44). Those aromatic compounds **201** could find use as fluorescent probes to identify the protein binding partner of a particular cyclohexadienal in studies of such compounds to activate neurite outgrowth activity [63]. The cyclohexadienal core **200** was generated by a cross-condensation reaction of α,β-unsaturated aldehydes mediated by L-proline (Scheme 44).

**Scheme 45:**
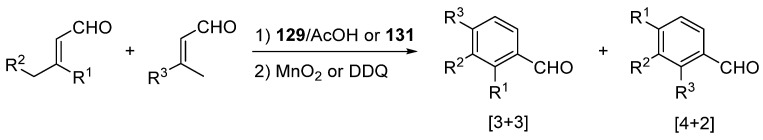
Organocatalytic synthesis of cyclohexadienals/oxidation reaction in the synthesis of aromatic aldehydes.

In line with this, the above reaction sequence of organocatalytic synthesis of the cyclohexadiene carboxaldehyde unit and subsequent oxidation reaction has been used as a fair strategy for the preparation of multi-substituted aromatic aldehydes [62]. Scheme 45 depicts a (4 + 2) and (3 + 3) cycloaddition of α,β-unsaturated aldehydes mediated by L-proline **131** or pyrrolide **129**/acetic acid (AcOH) to afford the cyclohexadiene carboxaldehyde intermediate that was not isolated and spontaneously oxidize or directly submitted to oxidation reaction conditions with MnO_2_ or DDQ for the formation of the aromatic compound [62].

**Scheme 46:**
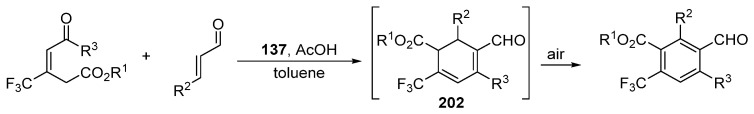
Organocatalytic synthesis of cyclohexadienals/aromatization reaction in the synthesis of pentasubstituted aromatic compounds.

In the following example (Scheme 46), pentasubstituted aromatic compounds are prepared under the above mentioned reaction sequence via intermediate cyclohexadiene carboxaldehyde **202** that was oxidized with atmospheric air appealing, therefore, in this case, under environmentally friendly reaction conditions [77]. Intermediate cyclohexadienal **202** is formed via a Michael type cascade reaction mediated by organocatalyst **137**.

### 3.2. Reactions Involving the Aldehyde Functional Group

**Scheme 47:**
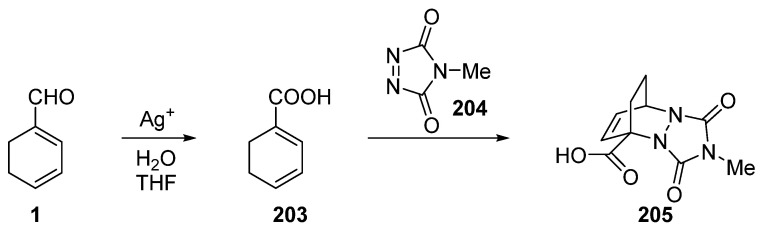
Oxidation of 1,3-cyclohexadien-1-carboxaldehyde to carboxylic acid **203**.

Oxidation of the aldehyde group in 1,3-cyclohexadien-1-carboxaldehyde to the carboxylic acid has been achieved with silver hydroxide prepared in situ (Scheme 47) [78]. Compound **203** was used in the cycloaddition reaction with 4-*N*-methyl-1,2,4-triazolin-3,5-dione **204**.

**Scheme 48:**
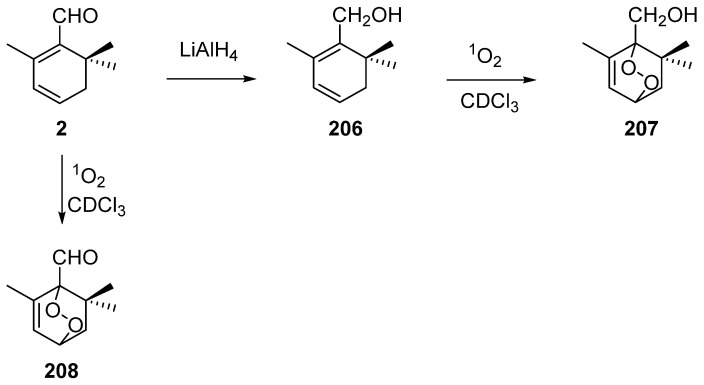
Reduction of safranal **2** to alcohol **206**.

Reduction reactions of the aldehyde group in the cyclohexadienal core have also been described. In this manner, the natural product safranal **2** was converted to the corresponding alcohol **206** in near quantitative yield with LiAlH_4_ (Scheme 48) [79]. The resultant alcohol **206** was submitted to a photooxygenation reaction for the formation of endoperoxide **207** in a chemoselective singlet oxygen (4 + 2) cycloaddition reaction. That transformation was also assayed directly on safranal compound **2**. However, the desired endoperoxide **208** turned out to decompose in this case.

**Scheme 49:**
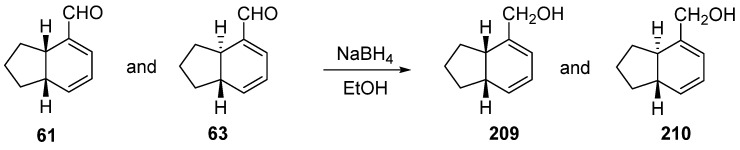
Reduction of natural aldehydes **61** and **63** to the corresponding alcohols.

Reduction of the aldehyde group in natural aldehydes **61** and **63** has been performed with the aim of confirming the structure of such natural compounds (Scheme 49) [6]. Aldehyde **63** present in black cardamom has been found to produce a trigeminal effect in the mouth.

**Scheme 50:**
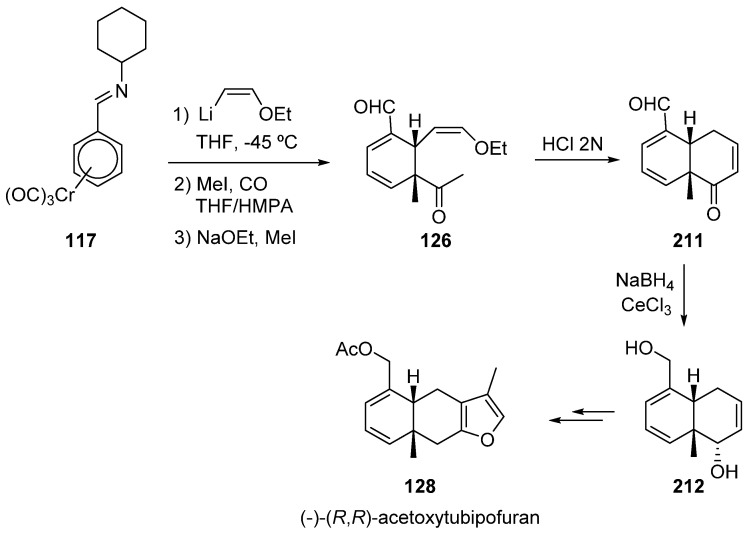
Reduction of the aldehyde moiety in the 1,3-cyclohexadien-1-al core in the total synthesis of acetoxytubipofuran.

The great versatility of the cyclohexadiene carboxaldehyde moiety is demonstrated in the utilization of such scaffold in the total synthesis of many varied compounds. That is the case of the citotoxic compound acetoxytubipofuran (Scheme 50). The strategy followed for its synthesis implies a tricarbonyl chromium-mediate dearomatization to access the substituted cyclohexadiene carboxaldehyde compound **126 [51]**. Further transformations to the final acetoxytubipofuran are followed. Among them, reduction of the aldehyde moiety in the cyclohexadienal core is performed with NaBH_4_ in the presence of CeCl_3_ (Scheme 50).

**Scheme 51:**
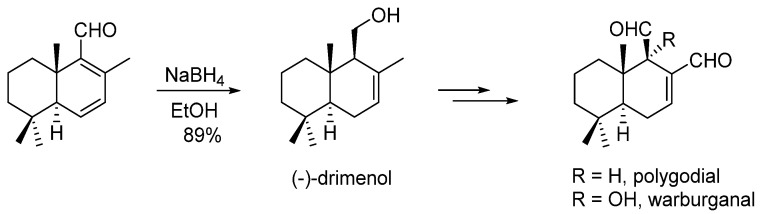
Reduction of the cyclohexadienal core in the synthesis of (−)-drimenol.

The synthesis of (−)-drimenol has also been achieved by means of a reduction reaction of the corresponding cyclohexadiene carboxaldehyde derivative (Scheme 51) [80]. Such reduction reaction unexpectedly involved the whole α,β-γ,δ-unsaturated aldehyde system. (−)-Drimenol, in turn, has been used as starting material in the synthesis of (−)-polygodial [81] and (−)-warburganal [82] (Scheme 51).

**Scheme 52:**
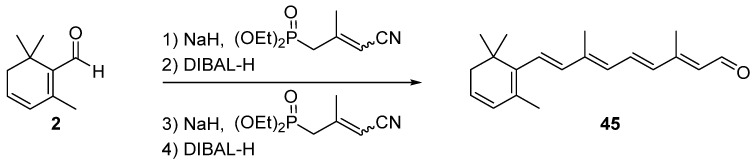
Horner–Wadsworth–Emmons reaction of safranal.

Another typical reaction of the aldehyde functional group is the Horner–Wadsworth–Emmons coupling with phosphonates [13]. Such transformation was the choice for the conversion of safranal **2** to 3,4-didehydroretinal **45** involving two consecutive phosphonate coupling reactions and reductions (Scheme 52).

**Scheme 53:**
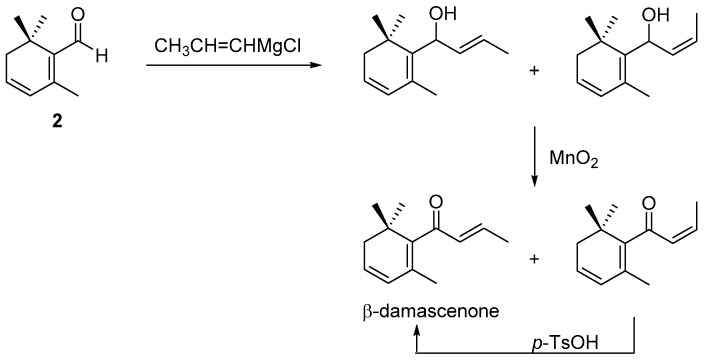
Reaction of safranal with a Grignard reagent.

Reaction of the aldehyde group with organometallic compounds has been used for the construction of miscellaneous structures. This is the case of the preparation of β-damascenone, wherein safranal **2** was reacted with the corresponding Grignard reagent (Scheme 53) [18]. Subsequent oxidation with MnO_2_ produced β-damascenone. Isomerization of the double bond on the side chain can be accomplished with *para*-toluensulfonic acid to obtain β-damascenone in higher yield (Scheme 53).

**Scheme 54:**
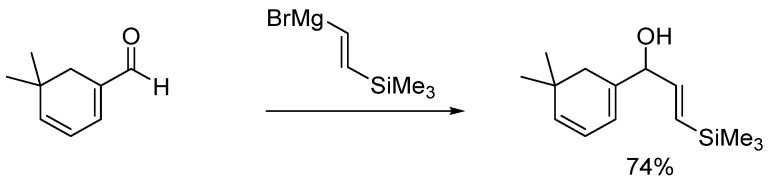
Reaction of cyclohexadiene carboxaldehyde unit with a Grignard reagent.

Scheme 54 shows the reaction of cyclohexadiene carboxaldehyde unit with another Grignard reagent bearing a silicon group [83].

**Scheme 55:**
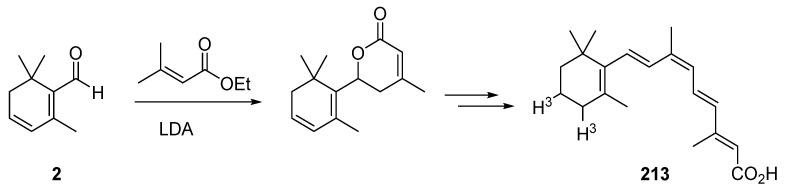
Reaction of safranal **2** with an organolithium reagent.

In the synthesis of tritium labelled 9-*cis*-retinoic acid **213** (Scheme 55), a key reaction of the cyclohexadienal core with an organolithium reagent was used in the initial stages [84].

**Scheme 56:**
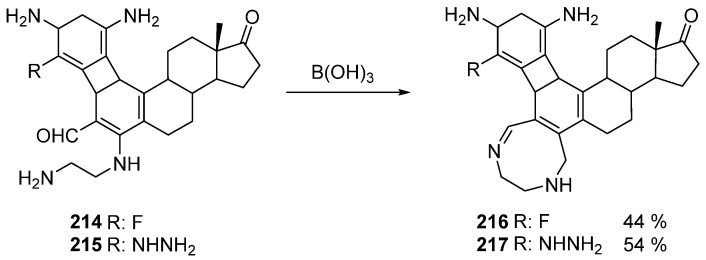
Intramolecular cyclization in the preparation of diazecine derivatives.

The aldehyde functional group has been harnessed in intramolecular (macro)cyclization reactions. Such is the case of the preparation of diazecine derivatives by intramolecular Schiff-base formation in the presence of boric acid (Scheme 56) [26].

**Scheme 57:**
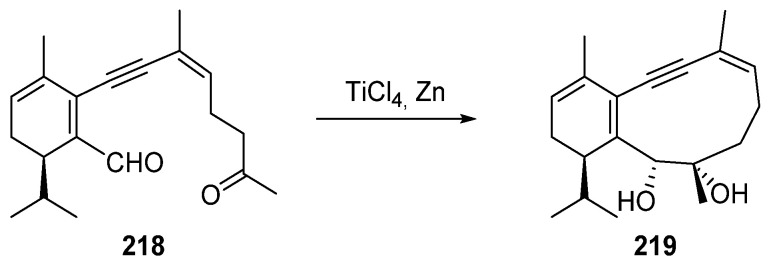
Intramolecular macrocyclization in the preparation of eunicellane skeletons.

The synthesis of diterpenoid eunicellane skeletons **219** incorporating 1,3-cyclohexadiene moiety is shown in Scheme 57, wherein a key step is the intramolecular pinacol macrocyclization reaction induced by low valent titanium under McMurry conditions [85].

**Scheme 58:**
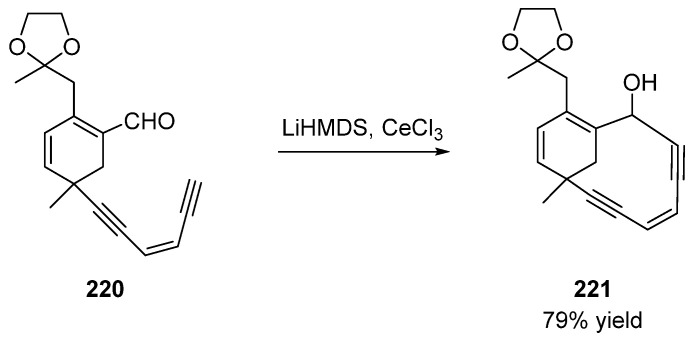
Intramolecular macrocyclization in cyclohexadienal derivative.

Another example of utilization of the aldehyde group of the cyclohexadienal core in intramolecular macrocyclization reaction is shown in Scheme 58 [86]. In this case, reaction of the aldehyde with the in situ-prepared organolithium reagent was accomplished using high dilution conditions in the presence of CeCl_3_.

### 3.3. Reactions Involving the α,β-Unsaturated Aldehyde System

**Scheme 59:**
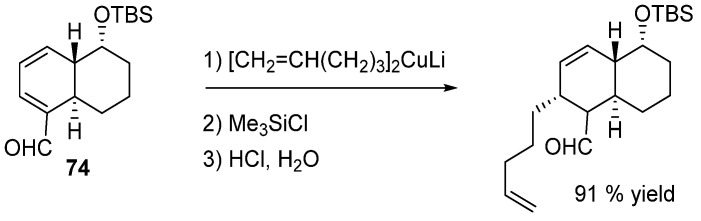
Organocuprate addition in cyclohexadienal derivative **74**.

Reactivity of the α,β-unsaturated aldehyde system has been utilized in the preparation of highly elaborated compounds. As an example, reaction of cyclohexadienal derivative **74** with an organocuprate is shown in Scheme 59, yielding mainly the 1,4-addition product [37].

**Scheme 60:**
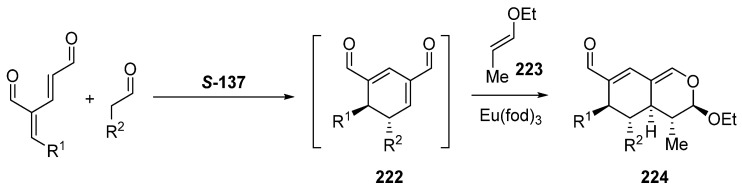
Inverse electron demand hetero-Diels–Alder reaction of cyclohexadienal derivative **222**.

The α,β-unsaturated aldehyde system in **222** can participate in an inverse electron demand hetero-Diels–Alder reaction with a vinylether **223** to form tetrahydroisochromene **224**, presenting five continuous stereocenters (Scheme 60) [68]. Such transformation has been achieved by means of the Lewis acid catalyst Eu(fod)_3_ [fod = 6,6,7,7,8,8,8-heptafluoro-2,2-dimethyl-3,5-octadienoate). In fact, the reaction is carried out in a one-pot fashion wherein organocatalytic annulation with formation of the cyclohexadiene dicarboxaldehyde **222** is followed by the hetero-Diels–Alder reaction by addition of the vinyl ether and the Lewis acid catalyst that should be compatible with the previous organocatalytic reaction conditions.

**Scheme 61:**
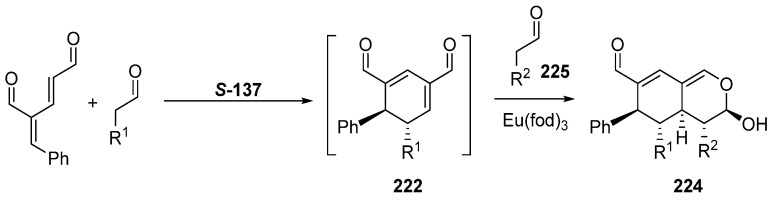
Cycloaddition reaction of cyclohexadienal derivative **222**.

Such a reaction concept has been extended further to achieve the sequential organocatalytic annulation reaction forming intermediate **222** and the subsequent cycloaddition reaction with a different aldehyde **225** (Scheme 61) [68]. In both cases, tetrahydroisochromenes **224** are formed in good yields and excellent stereoselectivities, and the great versatility of the cyclohexadienal core is highlighted.

## 4. Biological Activities of 1,3-Cyclohexadien-1-Carboxaldehyde

A variety of bioactivities have been reported for compounds containing the 1,3-cyclohexadienal scaffold: neuroprotective, anti-inflammatory, antibacterial, anticancer, cytotoxic, and phytotoxic.

**Figure 3:**
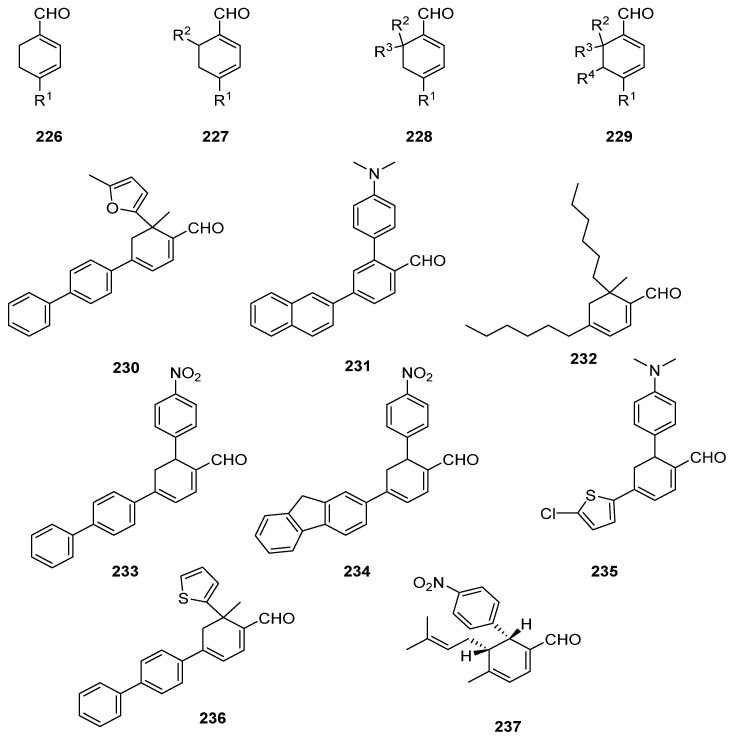
Heterodimeric and dimeric 1,3-cyclohexadien-1-als (**226**–**237**) and examples biologically active.

Apart from the organoleptic properties of *Vitis vinifera* that in part could be due to 1,3-cyclohexadien-1-carboxaldehyde, the main biological properties of this class of compounds have been studied in saffron. The saffron aqueous extract inhibits the chemically induced gastric cancer progression in the Wistar albino rat [87,88,89]. Safranal **2**, which is present in the flowers of *Crocus sativus* L., has been studied profusely, and many biological activities have been found for it. Safranal **2** has been proved to be a neuroprotective and anti-inflamatory agent [90,91,92,93,94,95,96,97,98,99]. Recently, Prof. Watanabe and colleagues have been involved in the study of substituted heterodimeric compounds generated by proline-mediated condensation of *α*,*β*-unsaturated aldehydes [53] (Figure 3).

Prof. Watanabe and colleagues evaluated the library of compounds for their ability to stimulate neurite outgrowth in a PC12 assay [63]. Two heterodimers, **230** and **231**, were able to stimulate neurite outgrowth, while homodimer **232** supported the development of neural extensions. It is interesting to highlight that the same compound **230** together with **233**–**235** demonstrated complete inhibition of cell growth at 1μg/mL against Jurkats, a human T cell leukemia cell line. Two compounds, **236** and **237**, were reasonably active against *Bacillus subtilis*, with MIC values within the 10–25 μg/mL range.

Recent studies with *Crocus sativus* L. (saffron) extract as well as its main constituents—crocin, crocetin, and safranal **2**—show interesting anticancer properties (Table 2). However, more studies are needed in order to evaluate the real single effect of each component, as in the combined treatment they may act synergistically against malignant cells. The in vitro results for safranal **2** showed a positive effect in leukemia and breast cancer and is a promising agent for treatment, prevention, and combined therapy for liver cancer [11].

The 1,3-cyclohexadienals such as safranal **2** are the direct precursors of endoperoxides such as **208** that show antimalarial activity [15,79].

Compound **6**, when compared with Botrydial as a reference compound for phytotoxicity and antibiotic activity, showed slightly less activity for these bioassays [7,14,100]. However, **6** is more active than other structurally-related Botrydial derivatives; thus, the 1,5-dialdehyde moiety seems to be fundamental for keeping Botrydial activity [7]. The structure-activity relationship studies for Botrydial analogues showed that the higher Michael acceptor capacity, the lower antibiotic and phytotoxicicty activities.

The use of Indian fungi of *Parmelia genus* as a food supplement, aimed the study of *Parmelia perlata* crude extracts, showed antimicrobial and antifungal activity. Among the isolated compounds from *Parmelia perlata* extracts, **7** is present, showing potent antibacterial activity and moderate antifungal activity compared with the other compounds isolated from the extract [8].

The in vitro assays of compound **36**, which was evaluated on human keratinocyte NCT 2544 cell line and three different in vitro studies, showed higher cytotoxicity compared with its corresponding aromatized analogue. Cyclohexadienal **36** shows similar IC_50_ values compared with SDS (sodium dodecyl sulfate), IC_50_ = 0.12 mM and IC_50_ = 0.13 mM, respectively, and thus it can be considered a strong irritant [101].

Cyclohexadienal **238** shows potent antibiotic activity against methicillin-susceptible, methicillin-resistant, and tetracycline-resistant *Staphilococcus aureus* (MSSA, MRSA, and TRSA, respectively). The bactericidal activities (MBCs) were lower due to the oxygen bearing substituent proximal to C-3 [102].

## 5. Conclusions

The 1,3-cyclohexadien-1-al scaffold is a naturally occurring fragment with special interest due to its presence in safranal (*Crocus sativus*) or citral dimer (*Flustra foliacea*) with antioxidant, neuroprotective, and antiinflamatory bioactivities. Different synthetic 1,3-cyclohexadien-1-als have also shown important biological activities such as cytotoxic, phytotoxic, antibiotic, and activator of neurite outgrowth. In this manner, the synthesis of new 1,3-cyclohexadien-1-als is of interest in medicinal chemistry as a potential source of bioactive compounds.

The different synthetic methodologies that have been used to obtain this scaffold are functional group interconversion, isomerization, Vilsmeier–Haack formylation, aldol-type and Horner reactions, organometallic reactions and organocatalysis-mediated transformations. Among them, the methods affording chiral 1,3-cyclohexadienal are pericyclic reactions, organometallic, chiral phosphine Horner reactions, and organocatalytic procedures. The latter organocatalytic methodologies have been the most widely used for this purpose.

Furthermore, a great versatility has been shown for the 1,3-cyclohexadien-1-carbaldehyde unit as building block with its participation in many different reactions for the construction of a great variety of compounds.

In summary, this manuscript shows the importance of the 1,3-cyclohexadien-1-als in organic synthesis, not only as building blocks but also in their biological activities as key compounds of interest in medicinal chemistry.

## Figures and Tables

**Table 1:** Examples of cyclohexadienals obtained in the (4 + 2) cycloaddition of α,β-unsaturated aldehydes mediated by L-proline.

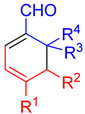					
**Compound**	**R^1^**	**R^2^**	**R^3^**	**R^4^**	**% Yield (ee)**
**50**	Me	H	H	Me	82 (--)
**150**	H	αOAc	αCH_2_OAc	H	68 (94)
**151**	H	H	αPh	H	61 (63)
**152**	Me	H	αPh	H	72 (32)
**153**	Me	H	αPh	H	78 (56)
**154**	Me	H	αEt	H	71 (--)
**155**	Me	H	αMe	H	82 (41)

**Table 2:** Biological activities of 1,3-cyclohexadien-1-als (**2**, **6**, **7**, **36**, **208**, **233**).

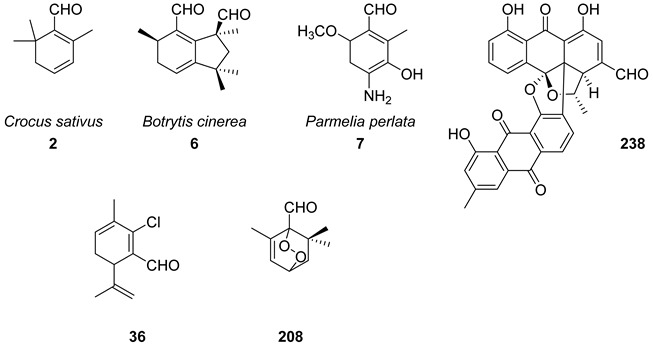			
**Compound**	**Activity**	**Test**	**Reference**
**2**	Anticancer, antioxidant, neuroprotective, anti-inflammatory	In vitro	[11,87,88,89,90,91,92,93,94,95,96,97,98,99]
**6**	Cytotoxic, phytotoxic, antibiotic	In vitro	[7,14,100]
**7**	Antibacterial, antifungal	In vitro	[8]
**36**	Cytotoxic	In vitro	[101]
**208**	Antimalaric		[15,79]
**233**	Antibacterial	In vitro	[102]
